# Supplementary material for: Control of capillary behavior through target-responsive hydrogel permeability alteration for sensitive visual quantitative detection
Source: Nat Commun. 2019 Mar 8;10:1036. doi: 10.1038/s41467-019-08952-1 (PMC6408548; doi:10.1038/s41467-019-08952-1)
Supplement: Supplementary file 1 — Supplementary Information [file 41467_2019_8952_MOESM1_ESM.pdf]

# Supplementary Information

Control of Capillary Behavior through Target-Responsive Hydrogel Permeability Alteration for Sensitive Visual Quantitative Detection

Li et al.

**Supplementary Table 1**. DNA sequences used for this work.

| name | sequences |
|---|---|
| Cocaine linker-Apt (L-Apt) | 5′-**ACT CAT CTG TGA** *ATC TCG GGA GAC* AAG GAT AAA TCC TTC AAT GAA GTG GGT CTC CC-3′ |
| Cocaine strand A (SA) | 5′-acrydite-AAA A**TC ACA GAT GAG T**-3′ |
| Cocaine strand B (SB) | 5′-acrydite-AAA A*GT CTC CCG AGA T*-3′ |

* Aptamer sequence highlighted with underline, and complementary sequences highlighted with boldface.

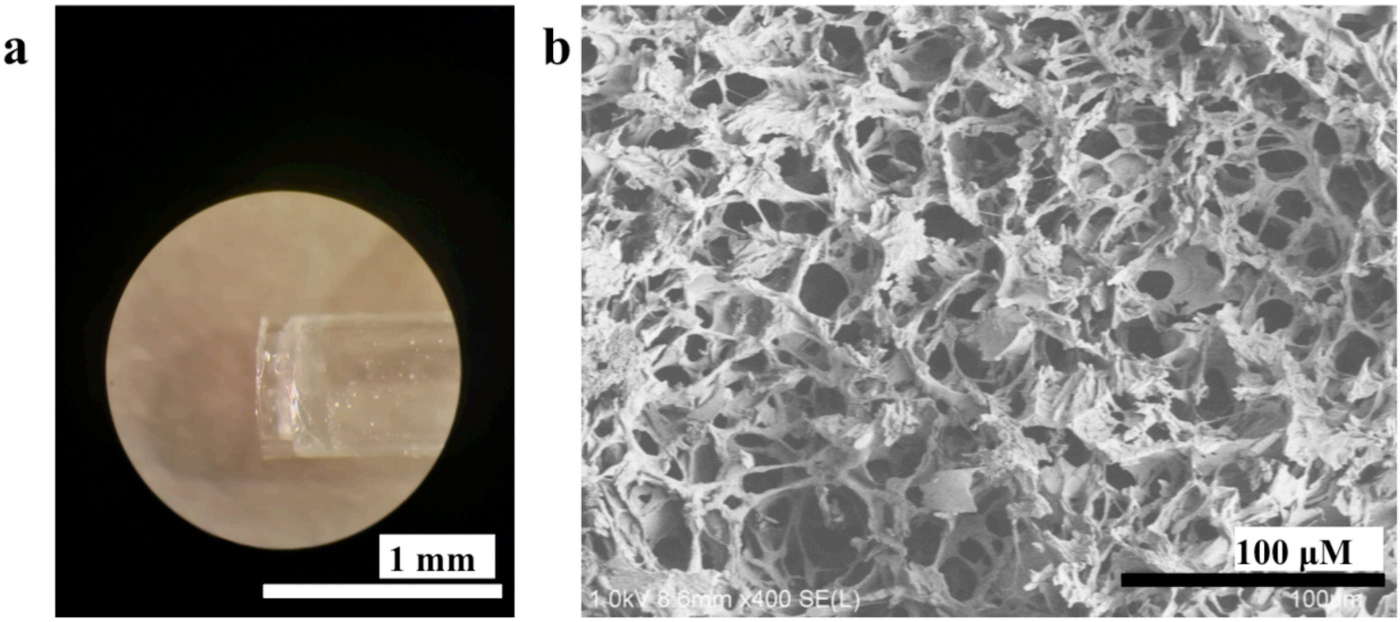


**Supplementary Figure 1.** Characterization of the DNA hydrogel. (a) The microscope photographs of DNA hydrogel film in capillary tube. (b) The high magnified SEM images of DNA hydrogel in capillary tube.

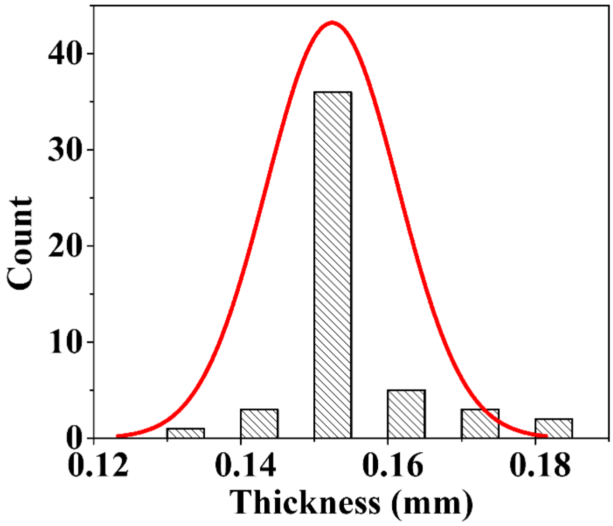


**Supplementary Figure 2.** Statistical data on the thickness of hydrogel film fabrication reproducibility (the statistical number is 50).

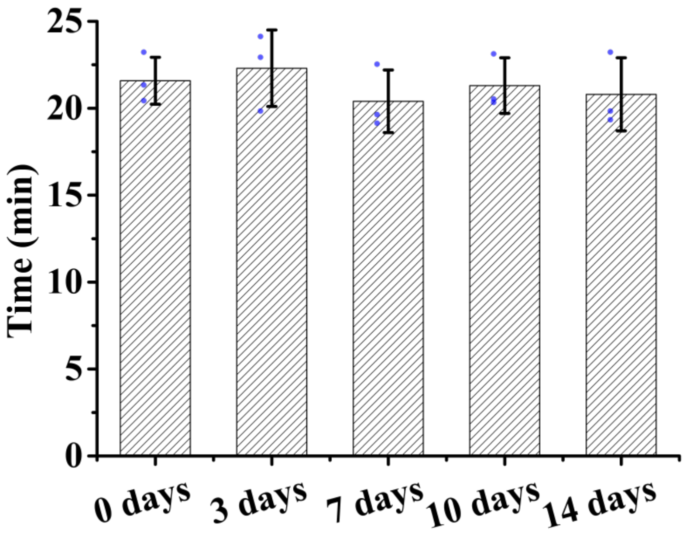


**Supplementary Figure 3**. The stability of the CSDR-Sensor has been tested at different times using 1 μM cocaine solution. Each data point is an average of three replicates, and the error bars indicate the standard deviations.

**Supplementary Table 2**. Performance comparison of this work with other assays base on DNA hydrogel.

| Detection method | Target | [a]LCR | [b]LOD | Gel volume | DNA Cost | Portable |
|---|---|---|---|---|---|---|
| Colorimetric[1] | Cocaine | semiquantitative | 5.9 μM | 10 μL | Very high | No |
| Colorimetric[2] | Cocaine | qualitative | —— | 10 μL | Very high | No |
| Glucose meter[3] | Cocaine | 0-750μM | 3.8 μM | 10 μL | Very high | Yes |
| Naked eye[4] | Cocaine | 0-400 μM | 0.06 μM | 10 μL | Very high | Yes |
| Naked eye[5] | Cocaine | qualitative | 50 μM | 1.5 μL | High | Yes |
| Naked eye[6] | Cocaine | 0-500 μM | 4.5 μM | 15 μL | Very high | Yes |
| UV-Vis spectra[7] | Cocaine | 0-2 mM | 15 μM | 10 μL | Very high | No |
| This method | Cocaine | 0-100μM | 1.17 nM | 0.01 μL | Very low | Yes |

[a]LCR, linear concentration range. [b]LOD, limit of detection.

## Supplementary References


[1] Zhu Z., Wu C., Liu H., Zou Y., Zhang X., Kang H., Yang C. J. & Tan W. An aptamer cross□linked hydrogel as a colorimetric platform for visual detection. *Angew. Chem.* **122**, 1070-1074 (2010).

[2] Yin B.-C., Ye B.-C., Wang H., Zhu Z. & Tan W. Colorimetric logic gates based on aptamer-crosslinked hydrogels. *Chem. Commun.* **48**, 1248-1250 (2012).

[3] Yan L., Zhu Z., Zou Y., Huang Y., Liu D., Jia S., Xu D., Wu M., Zhou Y. & Zhou S. Target-responsive "sweet" hydrogel with glucometer readout for portable and quantitative detection of non-glucose targets. *J. Am. Chem. Soc.* **135**, 3748-3751 (2013).

[4] Zhu Z., Guan Z., Jia S., Lei Z., Lin S., Zhang H., Ma Y., Tian Z. Q. & Yang C. J. Au@ Pt Nanoparticle

Encapsulated Target□Responsive Hydrogel with Volumetric Bar□Chart Chip Readout for Quantitative Point□of□Care Testing. *Angew. Chem. Int. Ed.* **53**, 12503-12507 (2014).

[5] Wei X., Tian T., Jia S., Zhu Z., Ma Y., Sun J., Lin Z. & Yang C. J. Target-Responsive DNA Hydrogel Mediated "Stop-Flow" Microfluidic Paper-Based Analytic Device for Rapid, Portable and Visual Detection of Multiple Targets. *Anal. Chem.* **87**, 4275-4282 (2015).

[6] Tian T., Wei X., Jia S., Zhang R., Li J., Zhu Z., Zhang H., Ma Y., Lin Z. & Yang C. J. Integration of target responsive hydrogel with cascaded enzymatic reactions and microfluidic paper-based analytic devices (μPADs) for point-of-care testing (POCT). *Biosens. Bioelectron.* **77**, 537-542 (2016).

[7] Mao Y., Li J., Yan J., Ma Y., Song Y., Tian T., Liu X., Zhu Z., Zhou L. & Yang C. A portable visual detection method based on a target-responsive DNA hydrogel and color change of gold nanorods. *Chem. Commun.* **53**, 6375-6378 (2017).
