# Supplementary material for: Control of capillary behavior through target-responsive hydrogel permeability alteration for sensitive visual quantitative detection
Source: Nat Commun. 2019 Mar 8;10:1036. doi: 10.1038/s41467-019-08952-1 (PMC6408548; doi:10.1038/s41467-019-08952-1)
Supplement: Supplementary file 3 — Description of Additional Supplementary Files [file 41467_2019_8952_MOESM3_ESM.pdf]

Description of Additional Supplementary Files

File Name: Supplementary Movie 1
Description: Performance of the CSDR-Sensor for the detection of 0 µM cocaine

File Name: Supplementary Movie 2
Description: Performance of the CSDR-Sensor for the detection of 1 µM cocaine

File Name: Supplementary Movie 3
Description: Performance of the CSDR-Sensor for the detection of 100 µM cocaine

File Name: Supplementary Movie 4
Description: Working principle of the CSDR-Sensor
